# Double-Drum Test Bench for Variable Load Transfer Simulation by Electromechanical Inertia Compensation

**DOI:** 10.3390/s19194322

**Published:** 2019-10-06

**Authors:** Zhichao Xing, Guoye Wang, Zhangpeng Gong, Shudong Zhang, Dongxin Xu, Sijie Peng

**Affiliations:** College of Engineering, China Agricultural University, Beijing 100083, China; guoye@cau.edu.cn (G.W.); gongzhangpeng@cau.edu.cn (Z.G.); zhangsd@cau.edu.cn (S.Z.); xudongxin1996@cau.edu.cn (D.X.); trepong@cau.edu.cn (S.P.)

**Keywords:** double-drum test bench, variable placement angle, load transfer, electromechanical inertia compensation, road test equivalence

## Abstract

To improve the accuracy and actual road equivalence of vehicle performance testing using test benches, a double-drum test bench that meets the test requirements of vehicle control system prototypes and in-use vehicles was designed. Dynamic models of the single-wheel test bench and the vehicle test bench were established, and mechanisms were theoretically analyzed for single-wheel variable adhesion and vehicle load transfer for equivalent testing using the variable placement angle. The mechanism of electromechanical inertia compensation was studied to realize stepless simulation of vehicle inertia and simulate dynamic load while braking. The simulation model of the vehicle test bench system was established based on MATLAB/Simulink. Simulations were carried out to verify the anti-lock braking system (ABS) performance test functionality of the test bench under high adhesion, bisectional, and low adhesion conditions. Referring to the simulation conditions, ABS tests under actual test bench and road conditions were carried out. Results demonstrated that the mechanism of variable load transfer simulation by electromechanical inertia compensation improves the equivalent accuracy compared to that of its road test equivalent, verifying the feasibility of the simulation mechanism. This study could help further improve the accuracy and reduce the cost of vehicle performance testing, thus greatly benefitting the vehicle development and testing industry.

## 1. Introduction

Vehicle performance testing is an effective method for evaluating the technical status of vehicles for both new vehicle development and testing in-use vehicles, which helps improve new vehicle development and reduces the cost of research and development. Braking performance is an important indicator of driving safety. Considering performance detection methods for conventional braking and the anti-lock braking system (ABS), some studies have analyzed real-time fault detection, identification, and isolation of ABS based on on-board sensor signals [1,2]. There are two main methods to test conventional braking and ABS performance using an external testing system; namely, road tests and bench tests. Road tests include the conventional braking test, emergency braking test, slop braking test, and road test with a variable adhesion coefficient. The road test method is accurate to real-world use and provides reliable results, but requires strict test site conditions, has a long testing life cycle and high cost, and is greatly affected by climate. Therefore, the road test method is suitable for inspection by vehicle sampling, but not for vehicle batch testing. The bench method includes a semi-physical simulation test bench and a vehicle test bench. Zhang developed an ABS hardware-in-the-loop (HIL) test bench to carry out real-time ABS control detection, using a servo motor to drive the gear ring of a real vehicle with ABS to generate a wheel speed signal [3]. Wu designed a pneumatic ABS controller based on fuzzy self-optimization, and added a braking torque feedback signal into the control system to achieve double closed-loop control of wheel angular velocity and braking torque for ABS performance testing on dSPACE [4]. An acceleration slip regulation (ASR) adaptive Proportion Integral Differential (PID) controller was designed based on throttle opening. Wang supplemented tire stress adjustment to this method, verifying the designed ASR algorithm by using the dSPACE HIL system [5]. An electric braking systems (EBS) controller was designed on an HIL test bench, and an electric dynamometer was used to simulate the load variation for the entire vehicle during the braking process. Conventional braking tests and emergency braking tests were carried out on this test bench to evaluate the effects of the designed braking control unit (BCU) [6]. Some studies were based entirely on semi-physical simulation bench testing to evaluate the controller effects of the designed ABS algorithm [7,8,9,10]. Semi-physical simulation provides a real-time coupling method between physical hardware and computer virtual simulation, providing a low test cost and good real-time performance, but the test accuracy is relatively low. Besides, while it is suitable for initial research of the control algorithm and prototype trial production, it is insufficient by itself. 

Research and development of vehicle test benches are currently underway in some universities and research institutes. The ABS test bench developed by Zhao and Hao can realize speedometer calibration, braking tests, and ABS performance tests. The state of sliding between road and tire were simulated by slip control of a torque loading device, and the variable adhesion condition between tire and road was simulated by changing the input exciting current of the magnetic particle clutch [11,12,13]. Most vehicle test bench and semi-physical simulation methods use magnetic particle clutches [11,12,13,14,15] and dynamic dynamometers to achieve simulation of different road adhesion conditions by changing the longitudinal force, which imposes some deficiencies, such as high cost, high energy consumption, and complicated calibration.

This paper proposes a simple and efficient method for changing the adhesive condition. Based on the variable placement angle, simulation of the variable adhesive condition can be achieved by changing the normal forces on tires. Road simulation with the variable adhesion condition can be realized in the single-wheel test and the simulation of load transfer during the braking process can be realized in the vehicle test, which can improve the accuracy of a bench test to be equivalent to a road test. The matching relationship was obtained based on theoretical analysis of the variable placement angle.

The double-drum test bench can realize the static detection of vehicle performance, so the bench needs to output kinetic energy through the rotating parts to be equivalent to the tested vehicle kinetic energy, after which it can meet the requirement of real-time dynamic load matching in the braking process. At present, the simulation of vehicle inertia on the test bench mainly adopts three methods: mechanical flywheel, electrical inertia, and electromechanical inertia compensation. Considering the nonlinear time-varying characteristics of vehicle inertia during the braking process, the mechanical flywheel has a huge structure and cannot realize stepless simulation of different test conditions. The compensation accuracy is related to the flywheel rank difference, and this method also has difficulties in assembly, disassembly, and maintenance. The electrical inertia simulation method can realize stepless inertia simulation, but when the target inertia is large, high motor performance and high energy consumption are required. In some papers, motor control and output were studied from the aspects of asynchronous motor modeling, rotational speed/torque vector controlling, simulation, and performance testing [16,17,18,19,20]. Electromechanical inertia compensation can cover the whole inertia interval, so as to ensure the accuracy of bench testing, but higher control accuracy is needed. Shuai proposed an inertia deviation control algorithm based on the train braking test bench. Empirical coefficients were introduced to fit the mapping relationship between the inertia deviation and the output torque of the motor. He designed an inertia simulation module and completed the preliminary verification test [21]. Liu designed a double input self-tuning fuzzy PID controller based on speed error and error rate to realize power test bench inertia self-compensation, and he simulated the designed self-tuning algorithm [22]. A semi-physical tracked vehicle test bench was designed based on dynamometer output dynamic load to simulate actual road load and vehicle inertia load. The robustness and adaptability of this test bench were verified through simulation testing [23]. A new electric vehicle test bench was designed based on an electric dynamometer to match vehicle inertia. Theoretical verification was carried out using a simulation model and an HIL test bench [24,25,26]. At present, the research on electromechanical inertia compensation are mainly based on simulations or semi-physical test benches, and there is no actual bench to verify the combined compensation effects. In this paper, based on the mechanical inertia of the flywheel, drums, and such other rotating parts, combined with the electrical inertia of the motor output, a stepless matching of the vehicle inertia could be realized by the test bench.

A double-drum test bench was developed in this paper based on the mechanism of variable load transfer simulation using electromechanical inertia compensation. Based on this low-cost and low-energy method, vehicle dynamic load could be simulated in a real time testing process, and the accuracy of the road test equivalence could be improved. Moreover, it was hoped that the bench test could output the evaluation results that were consistent with the road test. The developed bench provides the means for technical testing and detection for the whole development process of an active safety controller (ABS/ASR), from prototype development and algorithm testing to product performance testing. The test bench has the advantages of simplicity, high efficiency, low cost, and high accuracy equivalence.

The remainder of this paper is organized as follows. Section 2 gives an overview of the software and hardware system of the test bench. Section 3 establishes the dynamic models of the single-wheel test bench and the vehicle test bench based on variable placement angle and the electromechanical inertia compensation. Section 4 establishes the vehicle test bench system simulation model based on Matlab/Simulink, and simulations of conventional braking and emergency braking were carried out. In Section 5, we tested the conventional braking performance and ABS performance of a real vehicle on the test bench, and in Section 6, we carried out the tests under the actual road conditions. The last section provides concluding remarks.

## 2. Software and Hardware System of Test Bench

### 2.1. Hardware Structure of Test Bench

**Figure 1:**
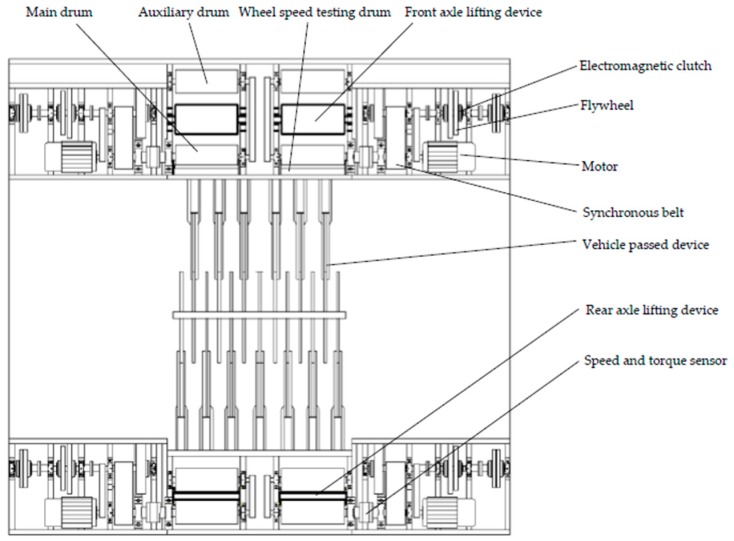
Structural diagram of double-drum test bench.

The structure of the double-drum test bench is shown in Figure 1. The test bench can perform the functions of speedometer calibration, single wheel/single axle/single side performance testing, vehicle conventional braking performance testing, and ABS performance testing. Before vehicle testing, the braking deceleration is set according to vehicle parameters and the test target to calculate load transfer. The placement angles between the wheels and two drums (main drum and auxiliary drum) should also be adjusted prior to testing. The test vehicle is then driven into the test bench and the front and rear axle lifting devices are lifted. The four wheels of the vehicle are weighed by pressure sensors installed on the lifting devices. Based on the calculation of equivalent inertia of each wheel, the necessary mechanical flywheels are mounted by combining corresponding electromagnetic clutches. The lifting devices are lowered while the wheel speed testing drums are lifted to contact each wheel. The speeds of the four wheels are detected in real time by the wheel speed sensors installed on the testing drums. The measured data of the speed sensors need to be converted by the ratio of the radius of the testing drum and the tested vehicle wheel to obtain the speed of the wheel. After the preparation work is completed, the tester can set user-defined test items and test procedures. The frequency converters are all set to speed control mode and drive the motors, driving the main drums and auxiliary drums and rotating the wheels. Speed and torque information regarding the main drums are collected real time by speed and torque sensors. When the speed reaches the preset initial braking speed, the control system prompts the driver to depress the brake pedal, and the frequency converters switch to torque control mode. The upper computer calculates the output torque of the four motors based on the compensation mechanism and sends control commands to the converters. Motors are controlled to output the matched torque, completing the electrical inertia simulation and realizing the stepless compensation of vehicle inertia. The test is stopped when the vehicle speed reaches 0 km/h, and the performance of the tested vehicle is evaluated according to the output test data of the measurement and control system. In single-wheel testing, a quarter-side bench can be driven by motor to reach the target speed, the braking system of the single-wheel device under testing is used to brake, and the electric inertia can be compensated in real time by switching the torque control mode of the frequency converter. The performance of single-wheel braking is tested and thus evaluated.

The placement of sensors on the electromechanical test bench is important, and there are some related studies on optimal sensor configurations. A new method to verify the matching state between the sensors and the system was proposed. Optimal sensor placement was achieved using the causal and structural properties of the bond graph tool, and the method was verified by an electromechanical test bench [27]. 

According to the functional requirements of the test bench, the combination of the quarter-side bench and the installation position of the main sensors is described as follows: the main-drum speed and torque sensor are mounted on the main transmission shaft; the wheel speed sensor is installed on the testing drum shaft; the flywheel speed sensors are installed on the corresponding positions on each flywheel; the pressure sensor is installed under the lifting device; the wheelbase displacement sensor is installed in between the front side and the rear side of the bench; and the vehicle in-place sensor is installed on the cover plate of the bench surface. The measurement and control function of the test bench is realized by configuring the sensors.

### 2.2. Measurement and Control System of Test Bench

**Figure 2:**
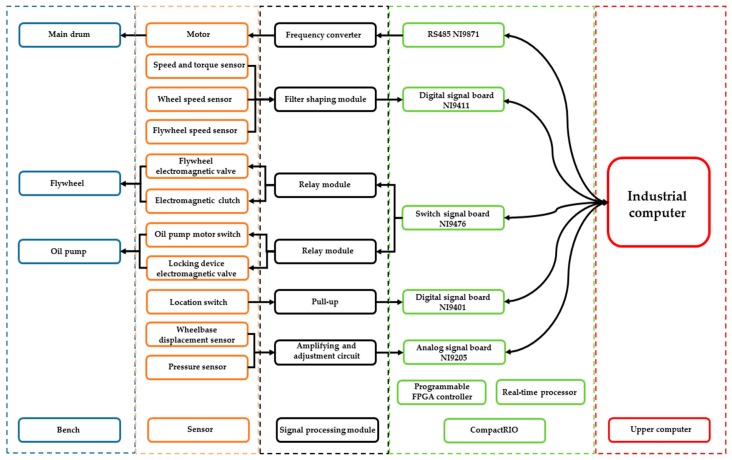
Framework structure of measurement and control system.

The double-drum test bench is an electromechanical–hydraulic integrated system, which has a variety of complex signals. It has a high demand for real-time I/O and expandable functions. The measurement and control system of the bench was designed based on system requirements. The system adopts a distributed structure based on the communication between the upper and lower computers, and the system adopts a master–slave cooperative working mode, which has high parallel processing efficiency and is convenient for system function expansion and maintenance. The industrial control computer communicates with the National Instruments (NI) CompactRIO embedded system based on Transmission Control Protocol/Internet Protocol (TCP/IP). CompactRIO includes a programmable Field Programmable Gate Array (FPGA) controller and a real-time processor, which can realize real-time signal processing and expand the functions of controller verification in the loop [28]. The inertia compensation function is realized by real-time communication between the NI communication board and the frequency converter. Sensor information output from the transmitting and conditioning module on the test bench are obtained by the NI Analog/Digital (A/D) board. All data are packaged and sent to the upper computer at regular intervals for real-time digital-curve display. The relay module controlled by the switch signal output board can make the corresponding bench actuators act according to given requirements. The overall structure of the measurement and control system is shown in Figure 2.

**Figure 3:**
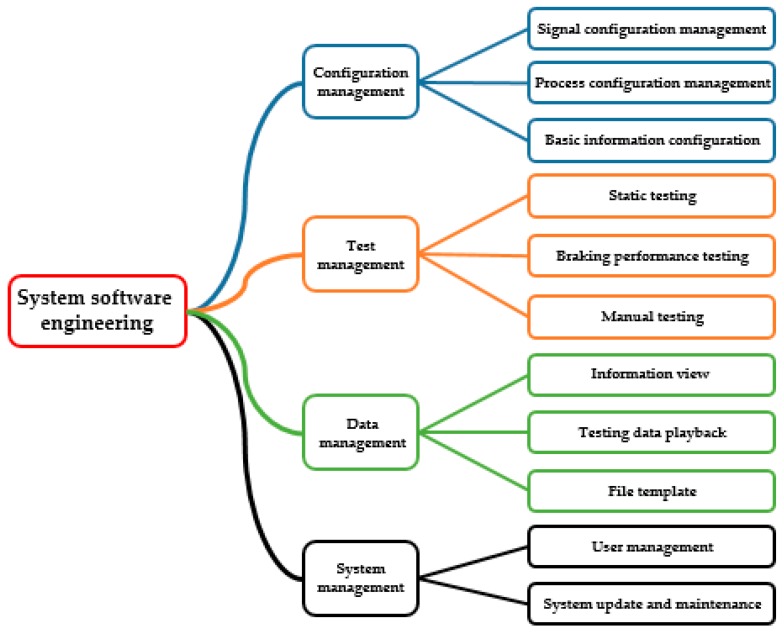
Structure of system software.

The multiform nested human–computer interaction software was written based on the Microsoft. NET framework development environment. The system software engineering includes configuration management, test management, data management, and system management. The framework of the software is shown in Figure 3. The software can realize the functions of basic information configuration, process and action management, self-testing of the measurement and control system, electromechanical inertia configuration, braking performance detection, data preservation, and test result output. It is convenient for users in the operation and observation of the system through customized visual configuration.

During the test, the measurement and control system collects real-time parameters such as the speed and torque of the four drums, pedal force, vehicle speed, and flywheel speed. The system then outputs performance evaluation indexes such as braking time, braking distance, slip rates, braking coordination time, and adhesion coefficient utilization, which can realize the conventional braking performance and ABS performance evaluation of the tested vehicle.

## 3. Variable Load Transfer Simulation by Compensation Electromechanical Inertia 

### 3.1. Variable Adhesion Simulation 

The simulation mechanisms were studied using the double-drum test bench. Placement angle refers to the angle between the vertical direction and the direction of the connecting line of the wheel center, and the contact point of the wheel and the drum.

#### 3.1.1. Simulation of Variable Adhesion Condition in Single-Wheel Test

**Figure 4:**
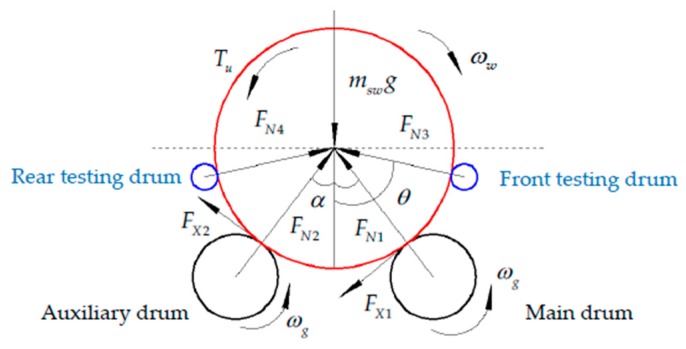
Analysis force of the single wheel.

Considering the wheel on the test bench, the wheel placed on the main drum and auxiliary drum is affected by the tangential forces and normal support reactions. In order to prevent the vehicle from driving out of the drum surface when braking, the wheel speed testing drums are placed in front and back of the wheel to assist in the safety of the testing. The force analysis is shown in Figure 4. The speed testing drums can rotate freely with little inertia, and the surface linear velocity can be approximated to the wheel speed. The tangential forces from the wheel speed testing drums acting on the wheel can be neglected.

Assuming that the testing drums are not subjected to force, the force analysis of the single wheel can be described as follows:

(1) $$\left\{ \begin{array}{l} (F_{N2}+F_{N1})\cos\alpha +(F_{X2}-F_{X1})\sin\alpha =m_{s}g\\ (F_{N2}-F_{N1})\sin\alpha -(F_{X2}+F_{X1})\cos\alpha =0 \end{array}\right.,$$

where $F_{N1}$ and $F_{N2}$ are the normal support reactions acting on the wheel by the main drum and auxiliary drum, respectively (N); $F_{X1}$ and $F_{X2}$ are the friction braking forces acting on the wheel by the main drum and auxiliary drum, respectively (N); $m_{sw}$ is the mass of the single wheel (kg); and α is the placement angle between the wheel and the two drums (rad).

It is known that the braking force coefficient varies with the slip rate in real time. Considering the condition of a dry pavement, the difference between the peak adhesion coefficient and the sliding adhesion coefficient is small. Assuming that the wheel is locked, the force analysis is described as follows:

(2) $$\left\{ \begin{array}{l} F_{X1}=\varphi_{g}F_{N1};F_{X2}=\varphi_{g}F_{N2}\\ (F_{X1}+F_{X2})=\frac{\varphi_{g}m_{sw}g}{(1+\varphi_{g}{}^{2})\cos\alpha } \end{array}\right.,$$

where $\varphi_{g}$ is the peak adhesion coefficient on the surface of the drum. As Equations (1–2) show, the simulation of variable adhesion condition can be realized by changing the placement angle between the two drums and the single wheel. The variable placement angle is realized by adjusting the distance between two drums, and the relationship can be obtained as follows:

(3) $$\mu^{\prime }=\frac{\varphi_{g}}{(1+\varphi_{g}{}^{2})\cos\alpha }=\frac{\varphi_{g}(r_{g}+R)}{(1+\varphi_{g}{}^{2})\sqrt{{\left(r_{g}+R\right)}^{2}-{\left(\frac{l_{g}}{2}\right)}^{2}}},$$

where $\mu^{\prime }$ is the equivalent peak adhesion coefficient; $l_{g}$ is the distance between the two drums (m); R is the wheel radius (m); and $r_{g}$ is the drum radius (m). Based on the equations mentioned above, the normal force acting on the single wheel can be changed by adjusting the placement angle, after which the function of multi-pavement test simulation in single-wheel tests can be realized.

#### 3.1.2. Simulation of Load Transfer during Braking in Vehicle Test

The influence of braking force distribution on directional stability and adhesion condition utilization in the vehicle braking test should be considered. At the road test site, the vertical load of each wheel under different braking forces of a commercial vehicle was tested, and the braking force distribution of the whole vehicle was evaluated [29]. Before analyzing the braking force distribution, the normal reaction force acting on each wheel in the braking process should be calculated. Due to the fact that the vehicle model is mainly used as the input of the test bench to verify the feasibility of the simulation mechanism, and considering the strong real-time performance required and the subsequent expansion of HIL function, the whole vehicle dynamics system is assumed and simplified as follows:

(1) Assuming that the rolling resistance and air resistance of wheels are fixed values;

(2) Assuming that the road surface is flat and the influence of slope is neglected;

(3) Assuming that the vehicle mechanism is rigid, the freedom of pitch and roll motion and the vertical freedom of four wheels are neglected. 

**Figure 5:**
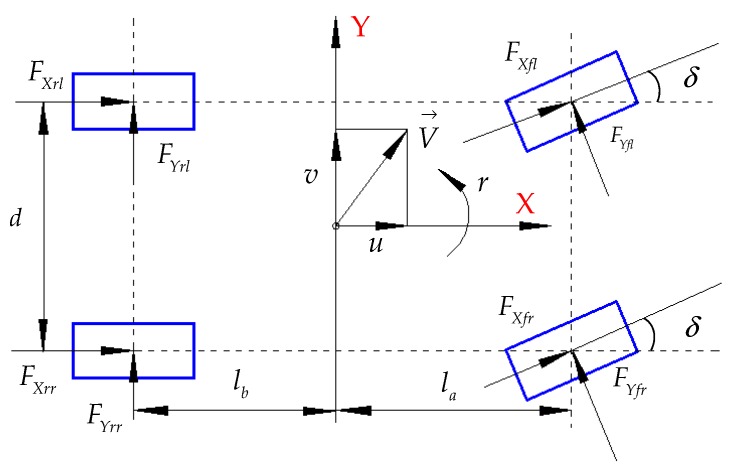
Analysis force of vehicle.

Based on the above assumptions and simplifications, the force analysis of the whole vehicle, considering load transfer, is shown in Figure 5.

The known vehicle driving equation is

(4) $$F_{t}=F_{f}+F_{w}+F_{i}+F_{j},$$

where $F_{t}$ is the driving force; $F_{f}$ is the rolling resistance; $F_{w}$ is the air resistance; $F_{i}$ is the grade resistance; $F_{j}$ is the acceleration resistance. The equation of rolling resistance and air resistance can be described as

(5) $$F_{f}+F_{w}=Gf\cos\alpha +\frac{C_{D}A}{21.15}V^{2},$$

where f is the rolling resistance coefficient; $C_{D}$ is the air resistance coefficient; and A is the frontal area. Considering that most test speeds in this research are about 50 km/h and the tests basically run in the ideal steady-state conduction, the values of these two parts are small and demonstrate slight change. These two parts were simplified as a fixed value into the specific testing data calculation. The dynamic model of a vehicle running on the horizontal road was established as shown below.

(6) $$\begin{array}{l} m(\overset{•}{u}-vr)=(F_{Xfl}+F_{Xfr})\cos\delta -(F_{Yfl}+F_{Yfr})\sin\delta +(F_{Xrl}+F_{Xrr})\\ m(\overset{•}{v}+ur)=(F_{Yfl}+F_{Yfr})\cos\delta +(F_{Xfl}+F_{Xfr})\sin\delta +(F_{Yrl}+F_{Yrr})\\ J_{z}\overset{•}{r}=l_{a}(F_{Yfl}+F_{Yfr})\cos\delta +l_{a}(F_{Xfl}+F_{Xfr})\sin\delta -l_{b}(F_{Yrl}+F_{Yrr})\\ -(\frac{d}{2})(F_{Xfl}-F_{Xfr})\cos\delta +(\frac{d}{2})(F_{Yfl}-F_{Yfr})\sin\delta -(\frac{d}{2})(F_{Xrl}-F_{Xrr}) \end{array}$$

where m is the vehicle mass (kg); u and v are the longitudinal and lateral velocity of the vehicle, respectively (m/s); r is the yaw rate of the vehicle (rad/s); $F_{Xfl}$, $F_{Xfr}$, $F_{Xrl}$, and $F_{Xrr}$ are the longitudinal forces on the left front, right front, left rear, and right rear tires, respectively (N); $F_{Yfl}$,$F_{Yfr}$, $F_{Yrl}$, and $F_{Yrr}$ are the lateral forces on the left front, right front, left rear, and right rear tires, respectively (N); δ is the front wheel angle (rad); $J_{z}$ is the inertia of the vehicle (kg·m^2^); $l_{a}$ and $l_{b}$ are the distances from the vehicle centroid to the center of the front and rear axles, respectively (m); d is the wheel track (m). The vertical loads of the four wheels can be described as follows:

(7) $$\left\{ \begin{array}{l} F_{Zfl}=\frac{mg}{\left(l_{a}+l_{b}\right)}(\frac{l_{b}}{2}-\frac{h_{g}\overset{•}{u}}{2g}-\frac{l_{b}h_{g}\overset{•}{v}}{gd})\\ F_{Zfr}=\frac{mg}{\left(l_{a}+l_{b}\right)}(\frac{l_{b}}{2}-\frac{h_{g}\overset{•}{u}}{2g}+\frac{l_{b}h_{g}\overset{•}{v}}{gd})\\ F_{Zrl}=\frac{mg}{\left(l_{a}+l_{b}\right)}(\frac{l_{a}}{2}+\frac{h_{g}\overset{•}{u}}{2g}-\frac{l_{a}h_{g}\overset{•}{v}}{gd})\\ F_{Zrr}=\frac{mg}{\left(l_{a}+l_{b}\right)}(\frac{l_{a}}{2}+\frac{h_{g}\overset{•}{u}}{2g}+\frac{l_{a}h_{g}\overset{•}{v}}{gd}) \end{array}\right.,$$

where $F_{Zfl}$, $F_{Zfr}$, $F_{Zrl}$, and $F_{Zrr}$ are the vertical loads on the left front, right front, left rear, and right rear wheels, respectively (N). The velocity of each wheel center along the direction of motion is described as follows:

(8) $$\left\{ \begin{array}{l} u_{fl}=(u-\frac{rd}{2})\cos\delta +(v+l_{a}r)\sin\delta \\ u_{fr}=(u+\frac{rd}{2})\cos\delta +(v+l_{a}r)\sin\delta \\ u_{rl}=u-\frac{rd}{2}\\ u_{rr}=u+\frac{rd}{2} \end{array}\right.,$$

where $u_{fl}$, $u_{fr}$, $u_{rl}$, and $u_{rr}$ are the center velocities of left front, right front, left rear, and right rear wheels (m/s). The slip rates of the four wheels can be described as follows:

(9) $$\left\{ \begin{array}{l} s_{fl}=1-\frac{R\omega_{fl}}{(u-\frac{rd}{2})\cos\delta +(v+l_{a}r)\sin\delta }\\ s_{fr}=1-\frac{R\omega_{fr}}{(u+\frac{rd}{2})\cos\delta +(v+l_{a}r)\sin\delta }\\ s_{rl}=1-\frac{R\omega_{rl}}{u-\frac{rd}{2}}\\ s_{rr}=1-\frac{R\omega_{rr}}{u+\frac{rd}{2}} \end{array}\right.,$$

where $s_{fl}$, $s_{fr}$, $s_{rl}$, and $s_{rr}$ are the slip rates of the left front, right front, left rear, and right rear wheels, respectively; $\omega_{fl}$, $\omega_{fr}$, $\omega_{rl}$, and $\omega_{rr}$ are the wheel angular velocities of the left front, right front, left rear, and right rear wheels (rad/s).

The UniTire tire model was selected for modeling [30]. The main equations are shown as

(10) $$\left\{ \begin{array}{l} s_{x}=-\frac{s_{i}}{1-s_{i}}\\ s_{y}=-\frac{\tan\theta_{i}}{1-s_{i}}\\ \phi_{x}=\frac{k_{x}s_{x}}{\mu_{x}F_{Zi}}\\ \phi_{y}=\frac{k_{y}s_{y}}{\mu_{y}F_{Zi}}\\ \phi =\sqrt{\phi_{x}{}^{2}+\phi_{y}{}^{2}} \end{array}\right.,$$

where $s_{x}$ and $s_{y}$ are the equivalent longitudinal slip rate and the equivalent lateral slip rate; $F_{Zi}$, $s_{i}$, and $\theta_{i}$ are the vertical loads, the slip rates, and the tire slip angles, i=(fl,fr,rl,rr); $k_{x}$ and $k_{y}$ are the longitudinal slip stiffness and the cornering stiffness; $\mu_{x}$ and $\mu_{y}$ are the longitudinal adhesion coefficient and the lateral adhesion coefficient; $\phi_{x}$, $\phi_{y}$, and ϕ are the relative longitudinal slip rate, the relative lateral slip rate, and the relative total slip rate. Dimensionless total shear force $\bar{F}$ and aligning arm of force $D_{x}$ can be described as follows:

(11) $$\left\{ \begin{array}{l} \bar{F}=1-\exp[-\phi -E_{1}\phi^{2}-(E_{1}{}^{2}+\frac{1}{12})\phi^{3}]\\ D_{x}=(D_{x0}+D_{e})\exp(-D_{1}\phi -D_{2}\phi^{2})-D_{e} \end{array}\right.,$$

where $E_{1}$ is the curvature factor of curve; $D_{x0}$, $D_{e}$, $D_{1}$, $D_{2}$ are the $D_{x}$-related factors. The expressions of the longitudinal force $F_{x}$, lateral force $F_{y}$, and the aligning torque $M_{z}$ are obtained as follows:

(12) $$\left\{ \begin{array}{l} F_{x}=\bar{F}\frac{\phi_{x}}{\phi }\mu_{x}F_{z}=\bar{F}\frac{k_{x}s_{x}}{\phi }\\ F_{y}=\bar{F}\frac{\phi_{y}}{\phi }\mu_{y}F_{z}=\bar{F}\frac{k_{y}s_{y}}{\phi }\\ M_{z}=F_{y}D_{x}=F_{x}F_{y}(\frac{1}{k_{cx}}-\frac{1}{k_{cy}}) \end{array}\right.,$$

where $k_{cx}$ and $k_{cy}$ are the longitudinal stiffness and the lateral stiffness. $k_{x}$, $k_{y}$, $\mu_{x}$, $\mu_{y}$, $k_{cx}$, $k_{cy}$, $D_{x0}$, $D_{e}$, $D_{1}$, and $D_{2}$ contain generation identification parameters. The introduction of relaxation length is of great significance to the unsteady state tire model, and the definition of the relaxation length in relation to cornering properties is shown as follows:

(13) $$l_{y}=k_{y}/k_{cy},$$

where $l_{y}$ is the cornering relaxation length. The tire force delay characteristic is expressed in the steady tire model and the lateral unsteady semiempirical model with wide application range is considered.

(14) $$k_{y}=\frac{dF_{y}}{ds_{y}},$$

(15) $$l_{y}=\frac{k_{y}}{k_{cy}}=\frac{dF_{y}}{ds_{y}}/k_{cy}.$$

The lateral slip rate can be obtained as:

(16) $$s_{y}+l_{y}\frac{ds_{y}}{dY}=s_{yn},$$

where $s_{yn}$ is the steady lateral slip rate. Lateral force after relaxation can be obtained. As for longitudinal-slip characteristic [30,31], longitudinal slip rate can be obtained as follows:

(17) $$s_{x}+l_{x}\frac{ds_{x}}{dX}=s_{xn},$$

where $l_{x}$ is the longitudinal-slip relaxation length; $s_{xn}$ is the steady longitudinal slip rate. Longitudinal force after relaxation can be thus obtained. 

After the vehicle model was completed, a dynamic model of the vehicle bench was established. The reactions of the drums supporting the tires can be changed by adjusting the placement angles between the two drums and each wheel, allowing the simulation of load transfer for the whole vehicle during braking. The corresponding relationship of the four wheel vertical loads, which are equivalent to the drum reaction forces of the bench, can be obtained as follows:

(18) $$\left\{ \begin{array}{l} \frac{Gl_{b}}{(l_{a}+l_{b})(1+\varphi_{g}{}^{2})\cos\alpha_{fl}}=\frac{G}{\left(l_{a}+l_{b}\right)}(\frac{l_{b}}{2}-\frac{h_{g}\overset{•}{u}}{2g}-\frac{l_{b}h_{g}\overset{•}{v}}{gd})\\ \frac{Gl_{b}}{(l_{a}+l_{b})(1+\varphi_{g}{}^{2})\cos\alpha_{fr}}=\frac{G}{\left(l_{a}+l_{b}\right)}(\frac{l_{b}}{2}-\frac{h_{g}\overset{•}{u}}{2g}+\frac{l_{b}h_{g}\overset{•}{v}}{gd})\\ \frac{Gl_{a}}{(l_{a}+l_{b})(1+\varphi_{g}{}^{2})\cos\alpha_{rl}}=\frac{G}{\left(l_{a}+l_{b}\right)}(\frac{l_{a}}{2}+\frac{h_{g}\overset{•}{u}}{2g}-\frac{l_{a}h_{g}\overset{•}{v}}{gd})\\ \frac{Gl_{a}}{(l_{a}+l_{b})(1+\varphi_{g}{}^{2})\cos\alpha_{rr}}=\frac{G}{\left(l_{a}+l_{b}\right)}(\frac{l_{a}}{2}+\frac{h_{g}\overset{•}{u}}{2g}+\frac{l_{b}h_{g}\overset{•}{v}}{gd}) \end{array}\right.,$$

where $\alpha_{fl}$, $\alpha_{fr}$, $\alpha_{rl}$, and $\alpha_{rr}$ are the placement angles between the two drums and the four wheels of the left front, right front, left rear, and right rear, respectively (rad). The corresponding relationship between the placement angles and braking deceleration of the vehicle can be described as follows:

(19) $$\left\{ \begin{array}{l} \cos\alpha_{fl}=\frac{2gdl_{b}}{\left(1+\varphi_{g}{}^{2})(gdl_{b}-dh_{g}\overset{•}{u}-2l_{b}h_{g}\overset{•}{v}\right)}\\ \cos\alpha_{fr}=\frac{2gdl_{b}}{\left(1+\varphi_{g}{}^{2})(gdl_{b}-dh_{g}\overset{•}{u}+2l_{b}h_{g}\overset{•}{v}\right)}\\ \cos\beta_{rl}=\frac{2gdl_{a}}{\left(1+\varphi_{g}{}^{2})(gdl_{a}+dh_{g}\overset{•}{u}-2l_{a}h_{g}\overset{•}{v}\right)}\\ \cos\beta_{rr}=\frac{2gdl_{a}}{\left(1+\varphi_{g}{}^{2})(gdl_{a}+dh_{g}\overset{•}{u}+2l_{a}h_{g}\overset{•}{v}\right)} \end{array}\right..$$

The simulation of vehicle load transfer while braking at a given deceleration can be realized by adjusting the placement angles, which can detect the braking force distribution of the four wheels during the braking process and evaluate the directional stability and utilization of the adhesion coefficient. Based on the equations mentioned above, it can be concluded that changing the normal reactions of drums on each wheel can be used with the variable placement angle to realize simulation of the variable adhesion condition in a single-wheel test as well as load transfer while braking in a vehicle test.

### 3.2. Electromechanical Inertia Compensation Mechanism

Considering the condition of the actual road used by the vehicle and ignoring the potential energy of the vehicle, the kinetic energy E of the vehicle includes the translational kinetic energy $E_{v}$ and rotational kinetic energy $E_{\omega }$, and the energy distribution of the vehicle is shown as follows:

(20) $$\left\{ \begin{array}{l} E=E_{v}+E_{\omega }\\ E_{v}=\frac{1}{2}mv^{2};\;\;E_{\omega }=\frac{1}{2}J\omega^{2} \end{array}\right..$$

The inertia factors are introduced to describe the energy distribution of the vehicle on the actual road surface [23], and the vehicle kinetic energy E can be described as follows:

(21) $$E=\frac{1}{2}J\omega^{2}+\frac{1}{2}mv^{2}=\frac{1}{2}m_{\delta }v^{2}=\frac{1}{2}(1+\delta_{1}+\delta_{2}i_{0}^{2}i_{g}^{2})mv^{2},$$

where J is the moment of inertia equivalent to the wheel (kg/m^2^); ω is the angular velocity of the wheel (rad/s); m is the total mass of the vehicle (kg); v is the vehicle speed (m/s); $m_{\delta }$ is the equivalent vehicle mass (kg); $\delta_{1}$ and $\delta_{2}$ are the factors of inertia; and $i_{g}$ and $i_{o}$ are the transmission ratios of the transmission and the main reducer, respectively. The equivalent output energy of the test bench can be obtained as follows:

(22) $$\frac{1}{2}(1+\delta_{1}+\delta_{2}i_{0}^{2}i_{g}^{2})m_{Zi}v^{2}=\frac{1}{2}J_{gi}\omega_{gi}{}^{2},$$

where $J_{gi}$(i=fl,fr,rl,rr) are the four parts of the bench equivalent to the moment of inertia at the main drums (kg/m^2^); $\omega_{gi}$(i=fl,fr,rl,rr) are the four main drums angular velocities (rad/s); and $m_{Zi}$(i=fl,fr,rl,rr) are the dynamic vertical loads of the four wheels (kg). The known dynamic vertical loads of the four wheels are shown as follows:

(23) $$\left\{ \begin{array}{l} m_{Zfl}=\frac{m}{\left(l_{a}+l_{b}\right)}(\frac{l_{b}}{2}-\frac{h_{g}\overset{•}{u}}{2g}-\frac{l_{b}h_{g}\overset{•}{v}}{gd})\\ m_{Zfr}=\frac{m}{\left(l_{a}+l_{b}\right)}(\frac{l_{b}}{2}-\frac{h_{g}\overset{•}{u}}{2g}+\frac{l_{b}h_{g}\overset{•}{v}}{gd})\\ m_{Zrl}=\frac{m}{\left(l_{a}+l_{b}\right)}(\frac{l_{a}}{2}+\frac{h_{g}\overset{•}{u}}{2g}-\frac{l_{a}h_{g}\overset{•}{v}}{gd})\\ m_{Zrr}=\frac{m}{\left(l_{a}+l_{b}\right)}(\frac{l_{a}}{2}+\frac{h_{g}\overset{•}{u}}{2g}+\frac{l_{a}h_{g}\overset{•}{v}}{gd}) \end{array}\right..$$

The angular velocities of the motor output shaft, flywheel shaft, and main drum shaft of the test bench can be described as follows:

(24) $$\left\{ \begin{array}{l} \omega_{g}=\frac{v}{r_{g}}\\ \omega_{e}=\omega_{f}=z_{f}\frac{v}{r_{g}} \end{array}\right.,$$

where $\omega_{g}$, $\omega_{e}$, and $\omega_{f}$ are the angular velocities of the main drum shaft, motor shaft, and flywheel shaft, respectively, (rad/s); $z_{f}$ is the transmission ratio of the main drum shaft to the flywheel shaft and the motor output shaft.

(25) $$J_{gi}=(1+\delta_{1}+\delta_{2}i_{0}{}^{2}i_{g}{}^{2})m_{Zi}r_{g}{}^{2}=J_{g}+J_{0}+z_{f}{}^{2}J_{f}+z_{f}{}^{2}J_{e}+J_{E},$$

where $J_{g}$, $J_{0}$, $J_{f}$, $J_{e}$, and $J_{E}$ are the moment of inertia of the drum group, the basic rotating parts, the flywheel, the motor rotor, and the equivalent inertia of the motor output torque, respectively (kg·m^2^). Based on Equations (22–25), the number of flywheels and the output compensation torque of the motor can be calculated. The output of the motor is shown as follows:

(26) $$T_{E}=\frac{J_{E}\frac{dw_{g}}{dt}}{z_{f}},$$

where $T_{E}$ is the compensation torque for the motor needing to be output (N·m). Due to the fact that deceleration in the braking process varies over time, it is necessary to collect the braking deceleration in real time for the calculation of dynamic load and the control of motor output electrical inertia to realize the compensation of electromechanical inertia. If the mechanical flywheel set of the test bench includes n gradient flywheels, the maximum translational mass based on the compensation of electromechanical inertia can be described as follows:

(27) $$m_{\max}=\frac{z_{f}{}^{2}J_{e}+J_{0}+J_{g}+J_{E\max}}{r_{g}{}^{2}}+\sum_{i=1}^{n}\frac{z_{f}{}^{2}J_{fi}}{r_{g}{}^{2}},$$

where $J_{E\max}$ is the maximum equivalent inertia of the motor output (kg·m^2^), and the compensation for the electric inertia of the motor should be satisfied as follows:

(28) $$J_{E\max}\geq \frac{1}{2}z_{f}{}^{2}J_{f\delta \min},$$

where $J_{f\delta \min}$ is the minimum inertia rank difference of the flywheel set (kg·m^2^). Based on Equation (28), the stepless simulation of inertia for the entire vehicle can be realized by the compensation of the test bench electromechanical inertia.

## 4. Simulation Verification

**Figure 6:**
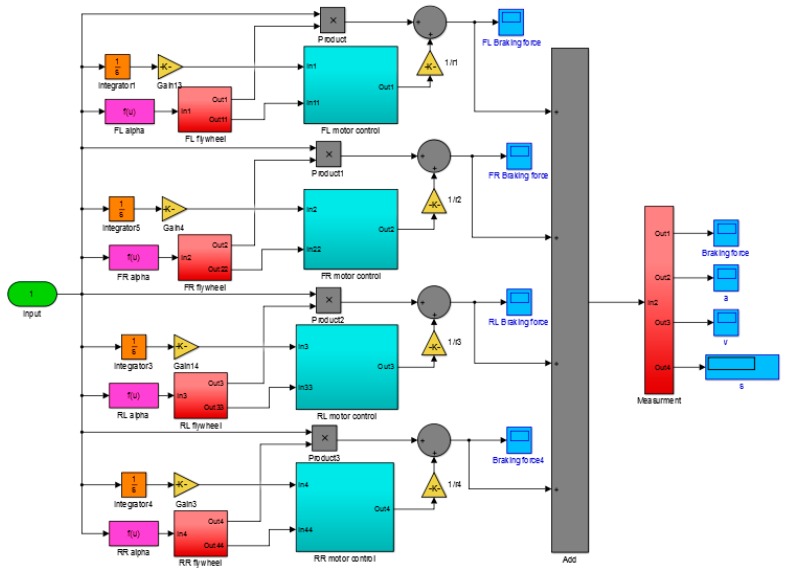
Vehicle test bench system simulation model.

The simulation of the vehicle test bench system is a nonlinear multibody dynamics problem. Aiming at nonlinear multibody dynamics problems, Briese et al. proposed a MODELICA-based multidisciplinary launch vehicle dynamics modeling, guidance, and control framework. Based on the framework proposed, applications such as trajectory optimization and nonlinear inverse model were implemented [32]. This method and its derived framework are also capable of solving full-vehicle multibody dynamics. Boreiry et al. established equations of motion based on the modified model for the Magnetorheological (MR) damper and built validation based on a 7-DOF vehicle model. Sensitivity analysis was performed based on MR damper to study the effects on vehicle response performance [33]. To verify the mechanism of variable load transfer simulation by electromechanical inertia compensation, a joint simulation model of the vehicle test bench system was established based on MATLAB/Simulink. This model considers whether the test bench can simulate the dynamic load in real-time through the simulation mechanism, and outputs the simulation results consistent with the pure vehicle simulation model. The model of the quarter-side test bench includes a variable placement angle module, a basic inertia module, a mechanical flywheel module, and a vector torque control module of the asynchronous motor. The whole vehicle simulation model was established based on the vehicle dynamics equations in Section 3. The system simulation model was then built by combining the whole vehicle dynamic model and the double-drum test bench model, as shown in Figure 6.

A light duty commercial vehicle was selected for simulation and the main parameters of the vehicle are shown in Table 1.

### 4.1. Conventional Braking Performance Simulation

**Figure 7:**
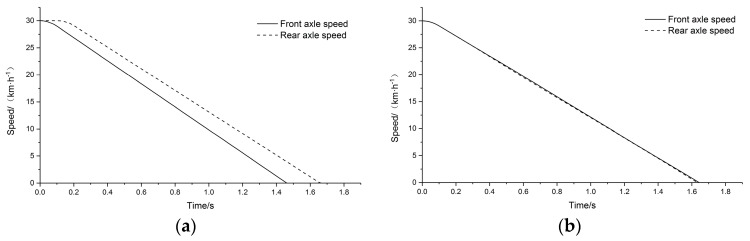
(**a**) Pure mechanical inertia; (**b**) electromechanical inertia compensation.

The output results of the system model under two simulation conditions, pure mechanical inertia compensation and electromechanical inertia compensation, were compared in the conventional braking performance simulation. After setting the appropriate initial speed and braking deceleration, the system simulation was carried out. The results are compared as shown in Figure 7.

Through this comparison, it can be seen that under pure mechanical inertia compensation, the rotational speed of the front and rear axle drums have a large deviation, and the equivalent speed of the main drums cannot be used, which greatly influences the braking performance evaluation of the vehicle. Under the electromechanical inertia compensation simulation, the rotational speed of the front axle drums is quite consistent with that of the rear axle drums, and the rotational speed of the main drums can reasonably be considered as equivalent to the vehicle speed. The conventional braking performance of the tested vehicle can be evaluated based on the vehicle test bench system.

**Figure 8:**
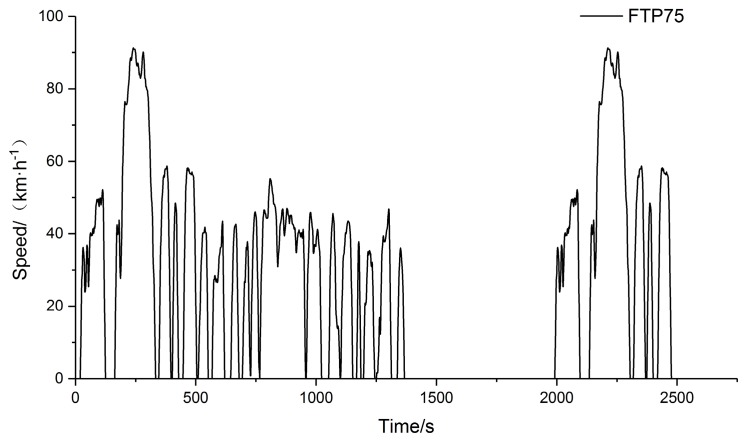
FTP75 cyclic condition.

The driving/braking torque output of the test system under a suitable cyclic condition was selected to verify the effects of the vehicle test bench system output as equivalent to driving conditions on actual road. Considering the application scenarios of the simulated vehicle, the FTP75 test condition was selected as the input condition for the simulation. The full cycle condition is shown in Figure 8.

**Figure 9:**
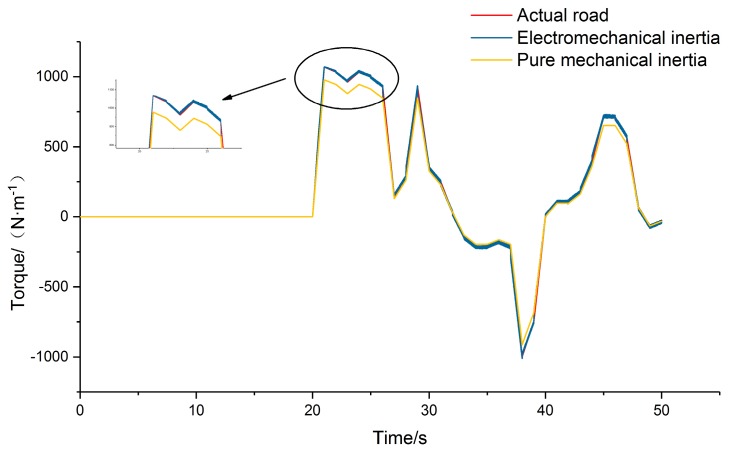
Comparison of driving/braking torque output.

The driving/braking torques of the tested vehicle under the FTP75 condition were compared under the actual road, pure mechanical inertia compensation, and electromechanical inertia compensation conditions of the test bench. Due to the large amount of simulation data, only the first 50 s cycle was selected for comparison. The maximum acceleration/deceleration value of the selected cycle section is |1.34|m/s^2^. The comparison of the output torque is shown in Figure 9.

Ignoring the influence of such factors as the internal friction in the bench and the friction of transmission parts, the simulation results show that the output driving/braking torque of the system under the pure mechanical inertia compensation condition has a larger error relative to actual road driving, with an average error close to 8.5%, greatly impacting the vehicle performance evaluation. The electromechanical inertia compensation condition can realize stepless inertia compensation of the vehicle. Compared with the actual road test simulation results, the average error is less than 2%. Therefore, the electromechanical inertia compensation mechanism can improve the feasibility and accuracy of the double-drum test bench to be equivalent to an actual road test.

### 4.2. ABS Performance Simulation

To verify the simulation mechanism, the placement angles between the two drums and each wheel were calculated and input into the model using appropriate braking deceleration. Tests were carried out for simulations of a high adhesion road, bisectional road, and low adhesion road under the condition of electromechanical inertia compensation simulation and the vehicle evaluation on the test bench was verified for ABS performance. 

#### 4.2.1. High Adhesion Condition

**Figure 10:**
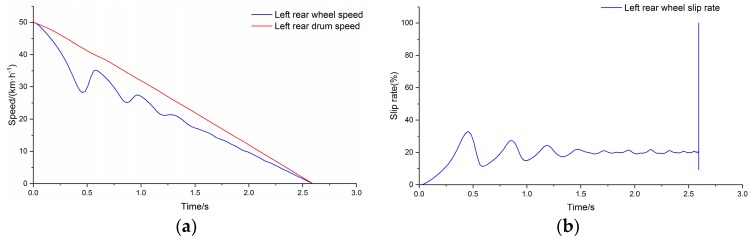
(**a**) Left rear comparison of main drum speed and wheel speed; (**b**) slip rate.

The commercial vehicle described in Table 1 was selected as the test vehicle. The ABS controller was established considering the ABS response and control characteristics of the test vehicle [34,35]. The peak adhesion coefficient of the drum surface was input to the test condition and the simulation was carried out. Figure 10a depicts the output of the simulation system for comparison of wheel speed and drum speed for the left rear side of the vehicle, and Figure 10b depicts the wheel slip rate calculated using the wheel speed and drum speed. The speed of the drum is used to represent the speed of the vehicle.

**Figure 11:**
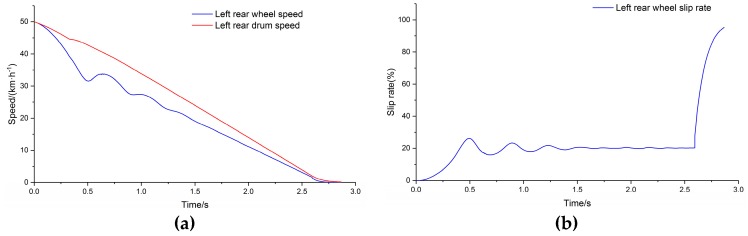
(**a**) Comparison of main drum speed and wheel speed; (**b**) slip rate after relaxation.

Figure 11 depicts the output of the simulation system after relaxation for the left rear side of the vehicle.

#### 4.2.2. Bisectional Condition

**Figure 12:**
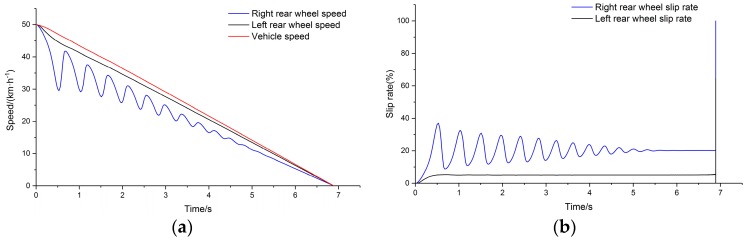
(**a**) Comparison of main drum speed and wheel speed; (**b**) slip rate.

The adhesion coefficient of the right drums were set as the peak adhesion coefficient after applying polytetrafluoroethylene (PTFE) tape. PTFE is an ideal material for low adhesion simulation because of its low friction coefficient and wear resistance, and high temperature resistance [36]. The running simulation model was completed after inputting the adhesion coefficient parameters. Figure 12a shows the comparison of the left and right rear side wheel speed and vehicle speed, and Figure 12b shows the comparison of the wheel slip rates.

#### 4.2.3. Low Adhesion Condition

**Figure 13:**
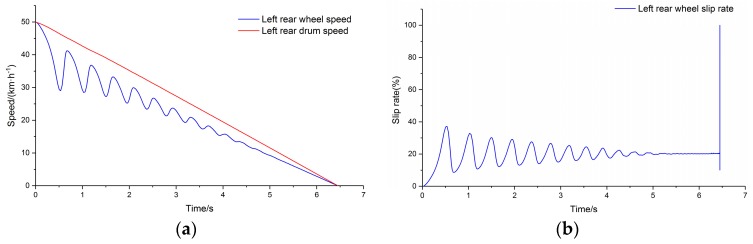
(**a**) Comparison of main drum speed and wheel speed; (**b**) slip rate.

The system model was simulated with the four parts of the simulation model all set to the surface peak adhesion coefficient of PTFE tape. Figure 13a shows the comparison of the left rear side wheel speed and drum speed, and Figure 13b depicts the wheel slip rate of the left rear side.

It can be concluded from the compared simulation results that the double-drum test bench based on load transfer simulation can realize stepless compensation of dynamic load while braking by the mechanism of electromechanical inertia compensation. The test bench can be used to evaluate the conventional braking and ABS performance of the vehicle, and the simulation conditions can be considered feasible as equivalent to a road test.

## 5. Test Bench Verification Test

**Figure 14:**
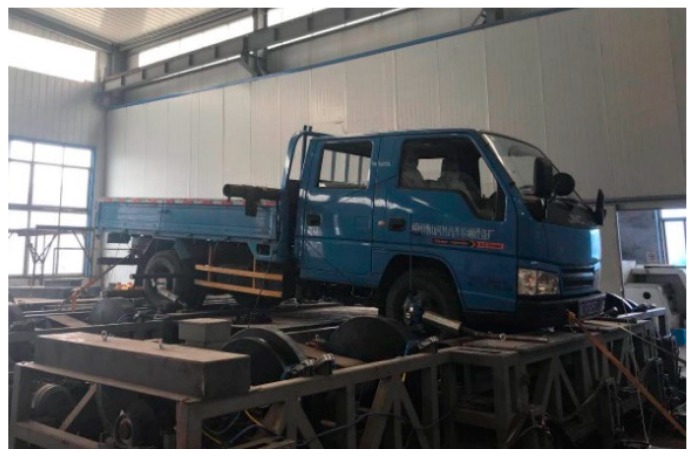
Vehicle test bench system.

The test system, as shown in Figure 14, employs the constructed hardware and software test bench and a commercial vehicle with parameters as listed in Table 1. The vehicle test bench system was capable of verifying the simulation mechanism while braking. Despite the brand-new high-performance bearings and related transmission components adopted in the test bench, considering the complexity of the transmission system, the tests of degraded operation are necessary in a later long-term continuous testing process. Benmoussa et al. proposed a hybrid estimation method based on remaining useful life (RUL). Dynamics modeling assumptions were made under normal operation, and the model parameters were identified based on sensor data. The model was then used to generate fault indicators and build a database for clustering [37]. For this test bench, sensors were equipped on the main transmission parts for identifying and detecting faults. Besides, the measurement and control system software have a corresponding monitor layer for real-time monitoring to ensure that the tests run normally.

### 5.1. Conventional Braking Performance Test

**Figure 15:**
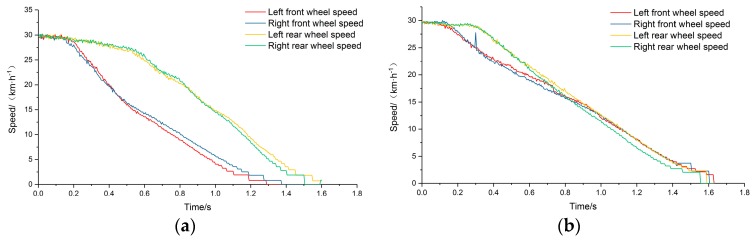
(**a**) Mechanical inertia compensation; (**b**) electromechanical inertia compensation.

In order to verify the mechanism of electromechanical inertia compensation, conventional braking performance tests were carried out. The outputs of the tests under pure mechanical inertia compensation and electromechanical inertia compensation were compared. The appropriate braking deceleration was set by adding a brake pedal position limiter to ensure that the wheels did not slip and lock in the braking process. The motors were switched to free parking mode and the pure mechanical inertia compensation test was carried out. The output speeds of the four main drums are shown in Figure 15a. Then, the electromechanical inertia compensation test was carried out. The motors were switched to torque output mode, with the limiter set in the same position. The braking deceleration was collected in real time to calculate the compensating torque of the four motors and the electric inertia was compensated by controlling the corresponding output command word of the frequency converters. The output results of the four main drums are shown in Figure 15b.

It can be seen by comparing the two tests that, under the condition of pure mechanical inertia compensation, the speed of the front and rear axle drums are clearly separated, so the vehicle speed cannot be characterized by the main drums, and parameters such as braking distance and braking time cannot be output to evaluate the conventional braking performance of the test vehicle. By comparison, under electromechanical inertia compensation, the four main drums are more suitable as parameters for calculating vehicle speed and evaluating the conventional braking performance of the test vehicle. Therefore, the electromechanical inertia compensation mechanism can improve test precision and ensure the accuracy of testing and evaluation.

### 5.2. ABS Performance Test

**Figure 16:**
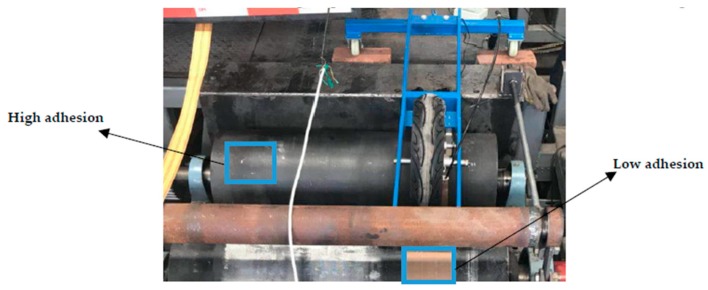
Adhesion coefficient test.

The ABS testing functionality of the bench was then validated. The placement angles between the two drums and wheels were adjusted according to the appropriate braking deceleration, and the load transfer during braking was simulated. The dynamic loads of the four wheels were compensated in real time. Comparative testing was carried out for the high adhesion, bisectional, and low adhesion conditions. The high adhesion condition was simulated by the drum surface, and the low adhesion condition was simulated by the drum surface with PTFE tape. The simulated surfaces with high and low adhesion coefficient were tested using a special adhesion determinator, as shown in Figure 16.

After many tests, the adhesion coefficient of drum surface was determined to be 0.78, and that of the PTFE tape surface was 0.27, which meet the requirements of the high adhesion and low adhesion tests. The parameters of adhesion coefficients in the simulation part were also input according to the test values in Section 4.

#### 5.2.1. High Adhesion Condition

**Figure 17:**
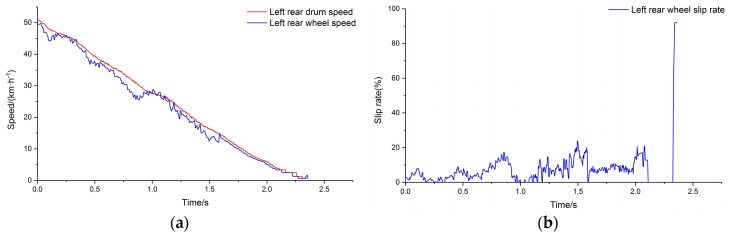
(**a**) Comparison of main drum speed and wheel speed; (**b**) slip rate.

The average braking deceleration result for the test vehicle was about 0.61 g when emergency braking was performed under the high adhesion condition. Then the no-load braking test under the high adhesion condition was carried out by adjusting the placement angles. The braking deceleration of 0.61 g was simulated, and the driver was prompted to depress the brake pedal for full braking when the speed reached 50 km/h. The left rear drum and wheel speeds were selected for analysis and comparison, and the slip rate of the left rear wheel was calculated. The test results are shown in Figure 17.

The test curves show that the ABS control effect of the vehicle is good under the condition of high adhesion when the wheels do not lock or slip in the process of braking. The results are listed in Table 2.

#### 5.2.2. Bisectional Condition

**Figure 18:**
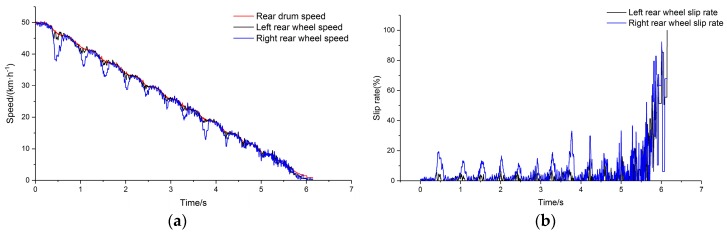
(**a**) Comparison of main drum speed and wheel speed; (**b**) slip rate.

PTFE tape was pasted on the right drums to simulate the bisectional condition test. The average braking deceleration of the vehicle was about 0.24 g under the bisectional test condition. The braking deceleration of 0.24 g was simulated by adjusting the placement angles. The wheel and drum speeds on the left and right sides of the rear axle were compared when the test was completed, and the slip rates were calculated. The test results are shown in Figure 18.

Vehicle braking under the simulated bisectional condition showed no obvious slippage on the wheels on the side with high adhesion. Speed fluctuation was on the side with low adhesion, and there was apparent ABS participation in the braking adjustment process. The results are listed in Table 2.

#### 5.2.3. Low Adhesion Condition

**Figure 19:**
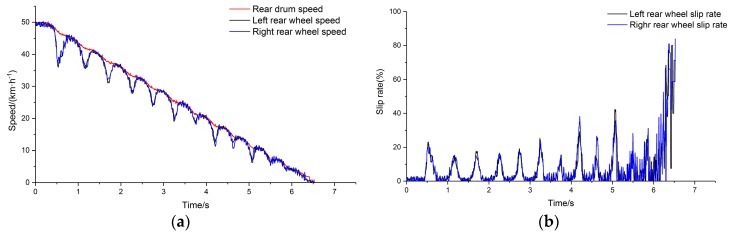
(**a**) Comparison of main drum speed and wheel speed; (**b**) slip rate.

PTFE tape was pasted on the left and right drums to simulate a single low adhesion condition. The average braking deceleration of the vehicle was about 0.21 g under this adhesion condition. The placement angles were adjusted based on 0.21 g braking deceleration. The speed of each wheel and drum on the left and right sides of the rear axle were compared, and the slip rates were calculated. The experimental results are shown in Figure 19.

Comparing the left and right side test curves under low adhesion conditions, the braking performance is shown to have good consistency, and the output results are listed in Table 2. The root mean square (RMS) and optimal proportion of the slip rate were calculated from the range of 40 km/h to 20 km/h deceleration.

The actual test environment is more complex than the simulation environment and there are some uncertain factors, such as unmodeled characteristics, disturbance, and friction. However, although the actual output results deviate from the simulation results, the overall trend is consistent. Based on the above test results for the high adhesion, bisectional, and low adhesion conditions, it can be concluded that the dynamic characteristics of the vehicle under different adhesion conditions and braking decelerations can be effectively simulated. Parameters such as vehicle speed, wheel speeds, braking time, and braking distance can be output, and the slip rates during braking can be calculated. Parameters, such as braking coordination time, mean fully developed deceleration (MFDD), and adhesion coefficient utilization can be used to evaluate the conventional braking and ABS performance of the tested vehicle.

## 6. Actual Road Verification Test

**Figure 20:**
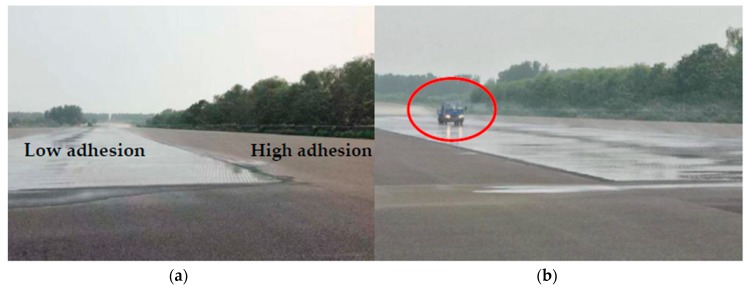
(**a**) Standard test pavement; (**b**) actual road test.

Pavement tests under the same test conditions were carried out on a standard high adhesion road, a bisectional road, and a low adhesion road in a proving ground. The standard pavement of the proving ground and road test are shown in Figure 20.

**Figure 21:**
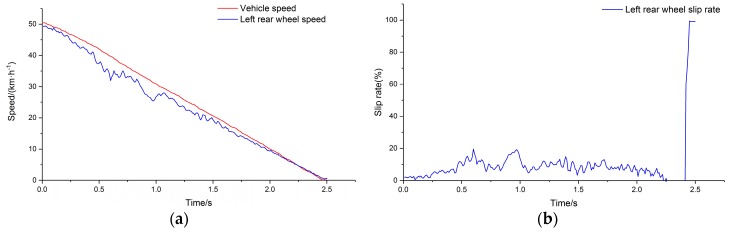

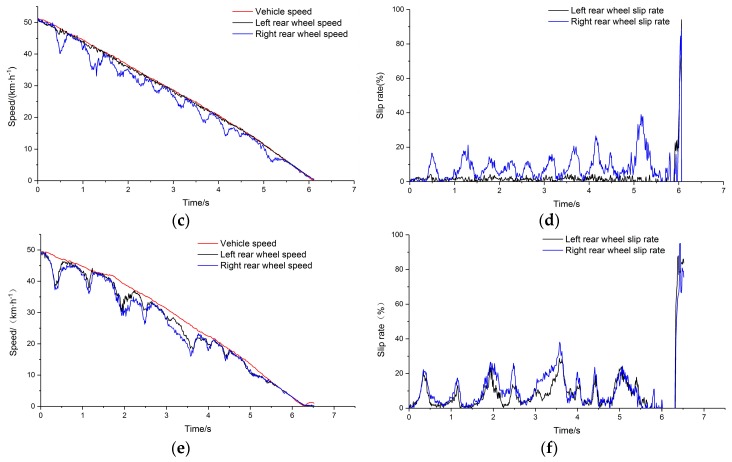
(**a**) Comparison of vehicle speed and speed of four wheels with high adhesion road; (**b**) slip rates with high adhesion road; (**c**) vehicle speed and speed of four wheels with bisectional road; (**d**) slip rates with bisectional road; (**e**) vehicle speed and speed of four wheels with low adhesion road; (**f**) Slip rates with low adhesion road.

The sensors were installed on the same test vehicle to collect real-time data and the output data were processed offline. The road test results are shown in Figure 21. From Figure 21, (a) and (b) are the output results of the high adhesion road, (c) and (d) are the output results of the bisectional road, and (e) and (f) are the output results of the low adhesion road.

The output evaluation parameters under different test conditions are shown in Table 3.

The output results of two tables were calculated, and the differences between the real test and the bench test are shown in Table 4.

This comparison shows that the error between the bench test and road test is noticeably smaller. The simulation mechanism is thus proven to be able to accurately simulate a real road test and improve the accuracy of conventional braking and ABS performance tests based on analysis of the experimental curves and parameter tables.

## 7. Conclusions

A double-drum test bench was designed based on the electromechanical integration of measurement and control that can satisfy development testing of ABS/ASR controller prototypes and the performance testing of in-use vehicles. The main conclusions are as follows:

(1) The dynamic model of the single-wheel test bench system was established and a theoretical analysis was carried out to simulate the equivalent peak adhesion coefficient of road surfaces through variable placement angles. A matching relationship between variable placement angle and equivalent peak adhesion coefficient was obtained, which can meet the requirements of single-wheel testing. The dynamic model of the vehicle test bench system was established based on the variable load transfer simulation while braking. The corresponding relationship between variable placement angles and variable load transfer simulation that could meet the requirement of vehicle testing was obtained.

(2) The mechanism of electromechanical inertia compensation was studied using the developed double-drum test bench. The stepless compensation of vehicle inertia was realized based on the compensation mechanism.

(3) A vehicle test bench simulation model was established based on MATLAB/Simulink. The characteristic parameters of the FTP75 condition were input to compare the torque output of the pure mechanical inertia compensation, the electromechanical inertia compensation, and the actual road test, which verified the consistency of the compensation mechanism with the actual road test. The ABS performance testing function of the test bench was tested by system simulation under the high adhesion condition, bisectional condition, and low adhesion condition, which verified the feasibility of the simulation mechanism for testing equivalence.

(4) Based on the developed test bench, pure mechanical inertia compensation and electromechanical inertia compensation were carried out to verify the electromechanical inertia compensation effect. Referring to the input conditions of simulation, ABS performance tests under high adhesion condition, bisectional condition, and low adhesion condition using the test bench and actual road testing were carried out, and the consistency of the comparison results was good.

The results show that the double-drum test bench can evaluate the conventional braking performance and ABS performance of the vehicle. The simulation mechanism by electromechanical inertia compensation while braking can improve the accuracy of testing. The test bench can provide results equivalent to the road test, which verifies the validity of the detection system and the simulation mechanism.

Considering the limitations of our study, all of the simulations and tests performed were based on a single type of commercial vehicle and no tests were carried out for different types of vehicles. Considering the diversity of vehicle types, it is necessary to test a variety of representative vehicles in the future. Characterizing the dynamic performance changes of the whole vehicle under some unsteady conditions through the test bench is also an important point and direction of our follow-up work. Taking other active safety technology performance testing into account, module function expansion should be added in the future to ensure the equivalency of the ASR performance test, Electronic Stability Program (ESP) performance test, and other functions. The cost of vehicle performance testing should be further reduced and the accuracy of equivalent testing should be improved by expanding our testing applicability.

## Figures and Tables

**Table 1:** Commercial vehicle parameter table.

Parameter	Value	Parameter	Value
Vehicle dimensions (mm)	5955 × 1780 × 2140	Type	Light truck
Curb weight (kg)	2460	Centroid height (m)	0.7
Wheelbase (m)	3.36	Distance between front axle and centroid (m)	1.595
Tire specification	7.00R16LT	Distance between rear axle and centroid (m)	1.765

**Table 2:** Output parameters of bench test.

Output Parameter	High Adhesion Road	Bisectional Road	Low Adhesion Road
Braking time (s)	2.37	6.02	6.71
Braking distance(m)	15.71	42.75	52.31
MFDD (m/s^2^)	6.26	2.45	2.27
Adhesion coefficient utilization	0.87	none	0.81
RMS of slip rate	9.87	none	7.57
Optimal proportion of slip rate (10%–20%)	28.21	none	21.71

**Table 3:** Output parameters of road test.

Output Parameter	High Adhesion Road	Bisectional Road	Low Adhesion Road
Braking time(s)	2.51	6.11	6.52
Braking distance(m)	17.52	46.64	49.43
MFDD(m/s^2^)	6.02	2.29	2.19
Adhesion coefficient utilization	0.82	none	0.85
RMS of slip rate	10.94	none	8.06
Optimal proportion of slip rate (10%–20%)	30.2	none	23.31

**Table 4:** Differences between real test and bench test.

Difference in Percentage (%)	High Adhesion Road	Bisectional Road	Low Adhesion Road
Braking time	5.57	1.47	2.91
Braking distance	10.33	8.34	5.51
MFDD	3.99	6.99	3.52
Adhesion coefficient utilization	6.10	none	4.71
RMS of slip rate	9.75	none	6.08
Optimal proportion of slip rate (10%–20%)	6.59	none	6.86
